# Molecular Diagnosis of Pathogenic *Sporothrix* Species

**DOI:** 10.1371/journal.pntd.0004190

**Published:** 2015-12-01

**Authors:** Anderson Messias Rodrigues, G. Sybren de Hoog, Zoilo Pires de Camargo

**Affiliations:** 1 Departamento de Microbiologia, Imunologia e Parasitologia, Disciplina de Biologia Celular, Universidade Federal de São Paulo (UNIFESP), São Paulo, São Paulo, Brazil; 2 CBS-KNAW Fungal Biodiversity Centre, Utrecht, The Netherlands; University of California San Diego School of Medicine, UNITED STATES

## Abstract

**Background:**

Sporotrichosis is a chronic (sub)cutaneous infection caused by thermodimorphic fungi in the order, Ophiostomatales. These fungi are characterized by major differences in routes of transmission, host predilections, species virulence, and susceptibilities to antifungals. *Sporothrix* species emerge in the form of outbreaks. Large zoonoses and sapronoses are ongoing in Brazil and China, respectively. Current diagnostic methods based on morphology and physiology are inaccurate due to closely related phenotypes with overlapping components between pathogenic and non-pathogenic *Sporothrix*. There is a critical need for new diagnostic tools that are specific, sensitive, and cost-effective.

**Methodology:**

We developed a panel of novel markers, based on calmodulin (*CAL*) gene sequences, for the large-scale diagnosis and epidemiology of clinically relevant members of the *Sporothrix* genus, and its relative, *Ophiostoma*. We identified specific PCR-based markers for *S*. *brasiliensis*, *S*. *schenckii*, *S*. *globosa*, *S*. *mexicana*, *S*. *pallida*, and *O*. *stenoceras*. We employed a murine model of disseminated sporotrichosis to optimize a PCR assay for detecting *Sporothrix* in clinical specimens.

**Results:**

Primer-BLAST searches revealed candidate sequences that were conserved within a single species. Species-specific primers showed no significant homology with human, mouse, or microorganisms outside the *Sporothrix* genus. The detection limit was 10–100 fg of DNA in a single round of PCR for identifying *S*. *brasiliensis*, *S*. *schenckii*, *S*. *globosa*, *S*. *mexicana*, and *S*. *pallida*. A simple, direct PCR assay, with conidia as a source of DNA, was effective for rapid, low-cost genotyping. Samples from a murine model of disseminated sporotrichosis confirmed the feasibility of detecting *S*. *brasiliensis* and *S*. *schenckii* DNA in spleen, liver, lungs, heart, brain, kidney, tail, and feces of infected animals.

**Conclusions:**

This PCR-based method could successfully detect and identify a single species in samples from cultures and from clinical specimens. The method proved to be simple, high throughput, sensitive, and accurate for diagnosing sporotrichosis.

## Author Summary

Sporotrichosis, a disease caused by infections from *Sporothrix* species, primarily affects warm-blooded animals, particularly humans and cats. Sporotrichosis is emerging as a global threat, with high incidences in Brazil and China. The gold standard for diagnosing sporotrichosis is microscopic characterization of the pathogen isolated in culture. This methodology is tedious and time-consuming. Moreover, closely related *Sporothrix* species are often misidentified, due to similar phenotypic characteristics. The introduction of dissimilar species with specific geographic distributions, host predilections, virulence, and antifungal susceptibilities, has made species-level identification of *Sporothrix* mandatory. To facilitate meeting this requirement, we developed a PCR-based method for detecting and identifying *Sporothrix* species. We designed species-specific primers for identifying *S*. *brasiliensis*, *S*. *schenckii*, *S*. *globosa*, *S*. *mexicana*, *S*. *pallida*, and *Ophiostoma stenoceras*. With this method, we could detect as little as 1 pg and 10 fg (depending on the species) of *Sporothrix* DNA derived from isolated cultures. Furthermore, we successfully detected *S*. *brasiliensis* and *S*. *schenckii* DNA in tissue samples derived from a murine model of disseminated sporotrichosis. These species-specific primers can be applied in epidemiology, clinical diagnosis, and experimental studies of sporotrichosis. Improvements in early diagnosis and surveillance systems may facilitate rapid identification and control of future outbreaks.

## Introduction

The fungal genus, *Sporothrix* [1–3], comprises a group of thermodimorphic fungi that cause human and animal sporotrichosis. In the saprophytic stage, thin hyphae with single-celled conidia form on clusters of denticles [4]. However, this morphology is observed in numerous species of Ophiostomatales, including *Sporothrix*, *Ophiostoma*, *Hyalorhinocladiella*, and *Pesotum* [4–10], and also in unrelated, clinically relevant fungi, such as the basidiomycete, *Quambalaria* [11].

Over the last decade, molecular studies on *Sporothrix* have revealed a clade of pathogenic species [12, 13]. In classical studies, Marimon *et al*. [13, 14] described *S*. *brasiliensis*, *S*. *schenckii s*. *str*., *S*. *globosa*, and *S*. *luriei*, which were mostly found in clinical samples. The phylogenetic reconstruction of *Sporothrix* demonstrated that the clade was distinct from *Ophiostoma* species, which has low potential of being pathogenic to mammals. *Sporothrix pallida*, *S*. *mexicana*, and the newly identified *S*. *chilensis* were nested in an environmental clade with *Ophiostoma*; these species live in association with soil, plant debris, and bark beetles [8, 10, 12].

Sporotrichosis is the most frequent (sub)cutaneous mycosis in urban areas [3, 15]. It affects both sexes, with no preferences for race or age. Despite its worldwide distribution, endemic areas are concentrated around temperate and tropical regions [16], and high incidences are found in Brazil, China, and South Africa [1, 15, 17, 18]. The relevance of identification at the species level has been underlined by genetic studies that recognized species differences by divergences in host associations, virulence and geographic distributions [9, 17, 19]. The classical species, *S*. *schenckii*, and the newly described species, *S*. *globosa*, are cosmopolitan pathogens that follow an environmental route of contamination via traumatic inoculation of contaminated decaying plant material [9]. At the other extreme, the highly virulent clonal offshoot, *S*. *brasiliensis*, is associated with animal infections and zoonotic transmission through deep scratches and bites from infected animals [3, 20], as well as it is associated with more severe clinical presentations in humans [21, 22].

Identification of *Sporothrix* species has key implications for the choice of antifungal therapy [23, 24] in different epidemiological risk groups [22]. Typically, *S*. *brasiliensis* shows the best response to antifungals *in vitro* [23–25] and *in vivo* [26, 27], whereas *S*. *schenckii*, *S*. *globosa* and *S*. *mexicana* may present the worst response [23, 24]. Incorrect identifications could potentially impact clinical outcome [23, 24]. Without appropriate treatment, the disease can evolve with severe manifestations in both humans and animals. Sporotrichosis infections persist for many months in cats, and outbreaks are intensified by cat-to-cat and cat-to-human transmissions [3, 17, 28]. Early diagnosis, surveillance, educational programs, and adequate treatment facilitate the containment and spread of the disease [29–31]. Early identification of etiological agents of sporotrichosis can be difficult, due to technical issues and the lack of affordable, effective methods. Rapid, reliable, low-cost *Sporothrix* detection methods are needed for adoption in endemic areas with resource-limited settings.

*Sporothrix* genotyping and diagnostics have focused on identifying *Sporothrix* genetic biomarkers, such as Random Amplified Polymorphic DNA (RAPD) [32, 33], Amplified Fragment Length Polymorphism [9, 34], PCR-Restriction Fragment Length Polymorphism (PCR-RFLP) [2], or protein fingerprinting, e.g., Matrix Assisted Laser Desorption Ionization Time-of-Flight [35]. Gold standard methods for species recognition are based on DNA sequences harbored in genomic loci that encode proteins, such as calmodulin [9, 13, 14], beta-tubulin [10, 13, 36], and translation elongation factor [9, 10, 17]. Additionally, the ribosomal internal transcribed spacer is recommended as a diagnostic marker for *Sporothrix* [3, 4]. Of the currently available diagnostic technologies, PCR-based methods predominate, because they typically have higher sensitivity and specificity across *Sporothrix* species compared to phenotypic tests [1, 37–39].

In the present study, we applied species-specific primers to (1) identify etiological agents of human and animal sporotrichosis in the *S*. *schenckii* clade; (2) enable large-scale screening of *Sporothrix* for epidemiological studies; (3) detect *Sporothrix* DNA in fresh tissue and biological samples from experimentally infected animals; and (4) highlight the utility of *Sporothrix* conidia as a simple, rapidly-processed source material for genotyping. A PCR-based system that targeted the calmodulin locus (*CAL*) was tested to fulfill these aims.

## Methods

### Ethics approval

This study was performed in strict accordance with recommendations in the Guide for the Care and Use of Laboratory Animals of the National Institutes of Health. The protocol was approved by the Ethics in Research Committee of the Federal University of São Paulo under protocol number 0244/11.

### Fungal strains and DNA extraction

Clinical isolates of *Sporothrix* were collected from patients with acute, lymphocutaneous or disseminated forms of sporotrichosis. Additional isolates were collected from felines and canines with sporotrichosis (S1 Checklist). *Sporothrix* and *Ophiostoma* isolates were obtained from the Federal University of Sao Paulo (UNIFESP), São Paulo, Brazil and CBS-KNAW Fungal Biodiversity Centre, Utrecht, The Netherlands (CBS). Isolates were stored as slant cultures on Sabouraud dextrose agar (Difco laboratories, Detroit, MI) at room temperature. These isolates were previously characterized at the species level by phylogenetic analysis of the *CAL* gene [1–3, 17]. Reference strains representing the main *Sporothrix* species were included in all experiments (Tables 1 and S1).

**Table 1:** *Sporothrix/Ophiostoma* strains used in this study for standardizing the PCR-based identification test.

Isolate code	CBS code	Species	Source	Origin	*CAL*	Reference
IPEC16490^T^	CBS 120339 ^T^	*S*. *brasiliensis*	Human	Brazil	12154878	[13, 14]
Ss54	CBS 132990	*S*. *brasiliensis*	Feline	Brazil	JQ041903	[1, 17]
Ss265	CBS 133020	*S*. *brasiliensis*	Human	Brazil	JN204360	[22]
CBS 359.36 ^T^	CBS 359.36 ^T^	*S*. *schenckii*	Human	USA	AM117437	[13, 14]
Ss118	CBS 132974	*S*. *schenckii*	Human	Brazil	JX077126	[1, 17]
Ss01	CBS 132961	*S*. *schenckii*	Human	Brazil	KC693828	[17]
FMR 8600 ^T^	CBS 120340 ^T^	*S*. *globosa*	Human	Spain	AM116908	[13, 14]
Ss06	CBS 132922	*S*. *globosa*	Human	Brazil	JF811336	[1, 17]
Ss49	CBS 132924	*S*. *globosa*	Human	Brazil	JF811338	[1, 17]
FMR 9108 ^T^	CBS 120341 ^T^	*S*. *mexicana*	Plant	Mexico	AM398393	[14]
Ss132	CBS 132927	*S*. *mexicana*	Human	Brazil	JF811340	[1, 17]
Ss133	CBS 132928	*S*. *mexicana*	Human	Brazil	JF811341	[1, 17]
CBS 302.73 ^T^	CBS 302.73 ^T^	*S*. *pallida*	Insect	UK	AM398396	[14]
PG3	-	*S*. *pallida*	Feline	Brazil	HQ404317	[40]
Ss329	-	*O*. *stenoceras*	Dog	Brazil	-	This study

IPEC, Instituto de Pesquisa Clínica Evandro Chagas, Fiocruz, Brazil; FMR, Facultat de Medicina i Ciències de la Salut, Reus, Spain; CBS, Centraalbureau voor Schimmelcultures, Utrecht, The Netherlands; ^T^, type strain. All “Ss” strains belong to the culture collection of Federal University of São Paulo (UNIFESP).

DNA was extracted and purified directly from fungal mycelial colonies with the Fast DNA kit protocol (MP Biomedicals, Vista, CA, USA) [1, 17]. DNA concentration was determined with a NanoDrop 2000 spectrophotometer (Thermo Fisher Scientific, USA), based on the default value, 1 OD (optical density) = 50 mg/mL double-stranded DNA; thereafter, DNA was diluted to a final concentration of 100 ng/μL. DNA quality was evaluated by determining ODs at wavelengths of 260 and 280 nm, and calculating the OD 260/280 ratio; only samples with OD 260/280 ratios between 1.8 and 2.0 were used in further analyses. The DNA samples were stored at -20°C until use in PCR reactions. Quality control for the DNA extraction process was assessed by amplifying part of the ribosomal DNA operon with the universal primers, ITS1 (5′-TCC GTA GGT GAA CCT TGC GG) and ITS4 (5′-TCC TCC GCT TAT TGA TAT GC) [41], as described previously [4]. Amplified products were evaluated with agarose gel electrophoresis. Amplification of a single product was regarded as a sample free of inhibitors.

### Gene sequences and primer design

Reference strains in the present study included the *CAL* gene nucleotide sequences from 277 isolates that belonged to the *S*. *schenckii* clade [3]. We also included *CAL* sequences from related species, genera, and other pathogens to increase the genetic diversity of our data-set and to cover most haplotypes described to date in the literature [3, 10, 17]. These sequences were previously deposited in GenBank and described by Marimon *et al*. [13, 14, 42], Rodrigues *et al*. [1–3, 10], Fernandes *et al*. [19], Sasaki *et al*. [40], and Zhang *et al*. [9]. The nucleotide sequences were aligned with the multiple sequence alignment software, MAFFT Version 7 [43]. Alignments of the *CAL* gene sequence were corrected manually with the software, MEGA 6 [44] to avoid mispaired bases. *CAL* sequences were used for *in silico* screening to identify parsimony informative sites that were either conserved or divergent intraspecifically, which could be used for primer design. Based on nucleotide polymorphisms, we manually chose short, informative regions with variations of 18–25 nucleotides. The software, Primer3 [45, 46] (http://primer3.wi.mit.edu/), was used to evaluate melting temperatures, %CG contents, dimer sequences, and mismatches in candidate sequences. Next, candidate primers were evaluated in Mfold software [47] for potential secondary structures, which could reduce amplification efficiency. Finally, we selected primers that would generate different product sizes for distinct species to facilitate identification with gel electrophoresis. Candidate primers were evaluated *in vitro* with conventional PCR, followed by gel electrophoresis and UV detection.

An internal positive control (IPC) that targeted the 5.8s region of the operon for the ribosomal DNA of *Sporothrix* and allied species, was introduced during PCR to check for the presence of fungal DNA. This positive control indicated whether a failure of amplification was due to PCR inhibition or due to a polymorphism present in the region targeted by the species-specific primers. IPC-forward and reverse primers (IPC-F and IPC-R, respectively) were used to genotype DNA that was purified directly from culture or DNA derived from conidia, as described below. However, these primers were not used for DNA detection in experiments conducted *in vivo* (experimental sporotrichosis).

### *In silico* PCR

Primers were further validated with *in silico* PCR; they were screened for specificity with the Primer-BLAST program [48] available at http://www.ncbi.nlm.nih.gov/tools/primer-blast/. Primer specificity was evaluated by using each primer pair to screen several genomic databases, included Ref-Seq mRNA, Genome (reference assembly from selected organisms and chromosomes from all organisms), and non-redundant databases, including Eukarya (taxid:2759), Fungi (taxid:4751), Bacteria (taxid:2), and Viruses (taxid:10239). Primer specificity stringency was set to recognize sequences with at least 2 total mismatches, to identify unintended, similar targets, and at least 2 mismatches had to appear within the last 5 bps at the 3' end. Targets that had 6 or more mismatches with a single primer were ignored.

### PCR optimization and gel electrophoresis

Total DNA extracted from *Sporothrix* or *Ophiostoma* was directly used as a template in PCR reactions for each candidate species-specific primer pair evaluated. The reactions were performed in a final volume of 25 μL, including 12.5 μL PCR Master Mix buffer (2×), which consisted of 3 mM MgCl_2_, 400 mM each dNTPs, and 50 U/mL Taq Polymerase (Promega Corporation, Madison, WI, USA); 9.5 μL water, 1 μL each of forward and reverse primers (10 pmol/μL; Integrated DNA Technologies, USA), and 1 μL of target DNA [100 ng/μL]. In PCR reactions with *Sporothrix* conidia, the water volume was adjusted to accommodate 0.5 μL of each IPC primer (IPC-F and IPC-R, each 10 pmol/μL). PCR reactions were performed in an Eppendorf Mastercycler Pro machine (Eppendorf, Hamburg, Germany). We used the touchdown PCR method to establish a block condition for the genotyping assay; i.e., a single program could be used for all primers (independently) at the same time. The conditions were as follows: an initial denaturing step of 5 min at 95°C; followed by 35 cycles of 1 min at 95°C, 1 min at the annealing temperature (touchdown PCR), and 1 min at 72°C; followed by a final step of 10 min at 72°C. In the touchdown protocol, the annealing temperature in the first cycle was 70°C, and subsequently, the annealing temperature was reduced by 1°C/2 cycles for the next 20 cycles; finally, the PCR was completed with an annealing temperature of 60°C for the remaining 15 cycles. PCR amplicons were resolved with 1.2% agarose gel electrophoresis for 1 h at 100V in the presence of GelRed (Biotium, Hayward, CA, USA). The stained bands were visualized with UV light with the L-Pix Touch (Loccus Biotecnologia, São Paulo, Brazil) imaging system.

### Assay specificity

For each primer pair (forward and reverse), we evaluated specificity with PCR amplification of DNA samples derived from clinical and environmental representatives of *Ophiostoma* and *Sporothrix* species. In addition, we tested several DNA samples derived from other pathogenic fungi, including agents of superficial, subcutaneous, and systemic mycoses in humans and animals. The PCR conditions and gel electrophoresis were described above.

### Assay sensitivity

We evaluated the sensitivity of each primer pair to ensure reliable amplification at low levels of the target DNA. We performed 10-fold serial dilutions of the DNA, starting with 100 ng/μL and ending with 0.01 fg/μL. The detection limit was noted for each primer pair.

### Non-target template competition

A pooled DNA sample (pool +) was created by mixing equal volumes of total DNA (100 ng/μL per strain) extracted from *S*. *brasiliensis* (CBS 120339), *S*. *schenckii s*. *str*. (CBS 359.36), *S*. *globosa* (CBS 120340), *S*. *mexicana* (CBS 120341), *S*. *pallida* (CBS 302.73), and *O*. *stenoceras* (Ss329). Then, 2 μL of this mixed DNA suspension was used as a target in PCR reactions, as described above. The specificity of amplification was confirmed by removing the target DNA sample from the pooled mixture (pool -) for each combination of primers.

### Molecular assay robustness

Despite their importance for inter-laboratory reproducibility, robustness and ruggedness are often neglected aspects of PCR-assay performance [49]. To evaluate different aspects of reproducibility, we assessed variations in primer concentrations (10–50 pmol/μL), template concentrations (100–500 ng/μL), and incubation times for annealing/extension temperatures (60–90 s). We also tested three PCR Thermal Cyclers, including the Eppendorf Mastercycler pro (Eppendorf, Germany), the Eppendorf Mastercycler ep Gradient S (Eppendorf, Germany), and the MG-96 MyGene Thermal Cycler (Long gene Scientific Instruments). With these assessments, we determined which features were most critical to inter-laboratory assay reproducibility. In addition, all experiments were repeated on different days, by investigators blinded to the identity of the tested samples and the results of other investigators.

### Experimental sporotrichosis

A systemic model of sporotrichosis was developed in 6–8 week-old male BALB/c mice, as described by Fernandes *et al*. [19]. Here, mice were infected with the highly virulent isolate of *S*. *brasiliensis*, CBS 132990, or with the moderately virulent isolate of *S*. *schenckii s*. *str*., Ss126 [19]. This model was used to evaluate the performance of the primers in detecting the two most common etiological agents of sporotrichosis [3, 17]. The animals were housed in temperature-controlled rooms at 23–25°C with *ad libitum* access to food and water. All the procedures were in accordance with the National Institutes of Health Animal and Care Guidelines. Every effort was made to minimize suffering.

Mice were divided into 3 groups of 5 animals each. Two groups were inoculated with *Sporothrix* (groups I and II) and one negative control group was inoculated with sterile phosphate-buffered saline (PBS) (S1 Flow Diagram). The mice were anesthetized with 0.2 mg/kg xilazine and 20 mg/kg ketamine. Then, animals were inoculated intravenously with 1×10^6^ yeast cells/animal (group I—CBS 132990, *S*. *brasiliensis*), 5×10^6^ yeast cells/animal (group II–Ss126, *S*. *schenckii s*. *str*.), or 100 μL sterile PBS (negative control group). Different concentrations were used, due to the variability in virulence between the different isolates [19]. Ten days post-infection, the animals were sacrificed with CO_2_ anesthesia, and the spleen, lungs, liver, kidneys, heart, brain, and tail were aseptically removed. The fungal burden in each organ was measured by quantitatively counting colony-forming units (CFU). Briefly, the organs were separated, weighed, and homogenized in sterile PBS with a tissue grinder. Samples (100 μL) of each homogenate were seeded on Petri dishes containing BHI agar and incubated at 37°C. Colonies were counted from the seventh day until the fifteenth day. The results were expressed as CFU/g tissue.

### Histopathological analysis

Tissue samples from infected groups were fixed in 10% formalin for 16 h, dehydrated in alcohol, and embedded in paraffin. Serial 5-μm sections were stained with Periodic acid-Schiff (PAS) to visualize the parasite morphology. Histological analyses of spleen, lungs, liver, kidneys, heart, brain, and tail were performed with an optical microscope (Olympus BX50, Japan) coupled to an Olympus DP71 CCD camera (Sony, Japan).

### Feces collection

Feces were collected from infected and control animals before euthanasia. Approximately 3–4 stools per animal were collected directly into sterile tubes. In addition, stool samples were collected after euthanasia from the rectum of animals. The samples were immediately processed after collection or stored at -80°C until use. Fecal DNA that originated from the host was extracted as described below.

### Fungal detection *in vivo* with PCR and species-specific primers

Fresh tissue fragments (~100 mg) from the five mice in each group were independently processed to extract DNA. Fragments of spleen, lungs, liver, kidneys, heart, brain, and tail were placed into screw cap tubes containing a 1/4-in diameter ceramic bead plus matrix A (MP Biomedicals, Vista, CA, USA). The specimens were frozen by incubating the tubes on dry ice for 10 min. The frozen tissues were homogenized in a Precellys 24-Dual homogenizer (Bertin Technologies, France) at 5000 rpm for 20 s; homogenization was repeated three times, with a 15-s interval between repetitions. Then, 1 mL of CLS-TC (Fast DNA kit—MP Biomedicals, Vista, CA, USA) was added, and the suspension was homogenized at 6500 rpm for 20 s; homogenization was repeated three times, with 10 s intervals between repetitions. DNA was purified according to the manufacturer’s instructions. DNA concentrations were determined with a NanoDrop 2000 spectrophotometer (Thermo Fisher Scientific, USA), and only samples with OD 260/280 ratios between 1.8 and 2.0 were used in subsequent assays.

Animal DNA quality was assessed by amplifying the β-actin gene in the BALB/c genome. We used the following primers: β-actin forward (5’-TCA CCC ACA CTG TGC CCA TCT ACG A) and β-actin reverse (5’-GGA TGC CAC AGG ATT CCA TAC CCA), as described by Pahl et al. [50]. These primers amplify a 347-bp fragment. β-actin PCR conditions were 95°C for 4 min; followed by 35 cycles of 1 min at 94°C, 1 min at 60°C, and 1 min at 72°C; and a final extension step of 10 min at 72°C. Products were analyzed with electrophoresis on 1.2% agarose gels and photographed under UV illumination. Samples that generated positive amplification signals were regarded to be free of PCR inhibitors.

DNA extracted from tissue samples was derived from both mouse and *Sporothrix* genomes. These DNAs were directly used as templates in PCR reactions with the primers, Sbra-F and Sbra-R or Ssch-F and Ssch-R, for each group. PCR reactions and cycling conditions were performed in a final volume of 25 μL, as described above (see PCR optimization and gel electrophoresis), except that we did not use the IPC. When the PCR assay resulted in negative or weak amplification, we took a sample from the PCR reaction solution (2 μL) and used it as a template for a second round of PCR (nested PCR) with the same primers.

### *Sporothrix* conidial PCR

*Sporothrix* cultures growing on the surface of SAB agar plates (5-cm diameters) were used for conidial PCR assays. We used type strain isolates of *S*. *brasiliensis* (CBS 120339), *S*. *schenckii* (CBS 359.36), *S*. *globosa* (CBS 120340), and *S*. *mexicana* (CBS 120341). Briefly, plates were incubated at room temperature for 3, 5, or 7 days. Approximately 300 μL of ultrapure water was added to the culture surface, and gentle swirling was applied to release the conidia. Typically, the resulting solution was clear and nearly invisible to the naked eye (at 520 nm, ODs were between 0.21 and 0.29). When the conidia were massively released, samples were diluted 1:1 in ultrapure water. Cells were transferred from the plates to a 200-μL tube, and disruption of *Sporothrix* conidia was achieved by heating the suspension for 10 min at 95°C in a thermal cycler. A sample (2 μl) was withdrawn and directly used as the template DNA in a PCR assay with species-specific primers. PCR conditions and electrophoresis were the same as described above, except that the initial incubation time at 95°C was increased from 5 min to 10 min, to improve cell lysis and DNA release. During standardization, the experiments were performed in duplicate to determine inter- and intra-assay variation. After standardization, experiments were performed with various clinical isolates, including *S*. *brasiliensis* (n = 15), *S*. *schenckii* (n = 15), *S*. *globosa* (n = 6), and *S*. *mexicana* (n = 4). As a positive control, we employed fungal DNA that was extracted with a conventional DNA preparation protocol.

### Statistical analysis

Diagnostic parameters included sensitivity, specificity, positive predictive value, and negative predictive value. Receiver operating characteristic (ROC) curves were prepared and analyzed to determine the sensitivity and specificity of each primer pair (Sbra-F+Sbra-R and Ssch-F+Ssch-R) for detecting *Sporothrix* infections (S1 Checklist). The area under the curve (AUC) in the ROC analysis was calculated to evaluate the diagnostic value of PCR and culture (CFU) methodologies. We assumed that a test lacked diagnostic power when the ROC curve was linear with an AUC of 0.5 (the ROC curve coincided with the diagonal). A test was assumed to have powerful diagnostic value when the ROC AUC was ~1.0, indicating the absence of both false-positives and false-negatives (the ROC curve reached the upper left corner of the plot). To measure the degree of concordance between the results from PCR and culture (CFU) and the results from PCR and phylogeny (*CAL* or ITS), we calculated the *Kappa* statistic and its 95% confidence interval (CI). *Kappa* values were interpreted as follows: 0.00–0.20, poor agreement; 0.21–0.40, fair agreement; 0.41–0.60, moderate agreement; 0.61–0.80, good agreement; 0.81–1.00, very good agreement [51]. *P*-values ≤0.05 were considered statistically significant. All calculations were performed with MedCalc Statistical Software, version 14.8.1 (MedCalc Software bvba; http://www.medcalc.org).

## Results

### Oligonucleotide design and *in silico* specificity of candidate primers

**Fig 1:**
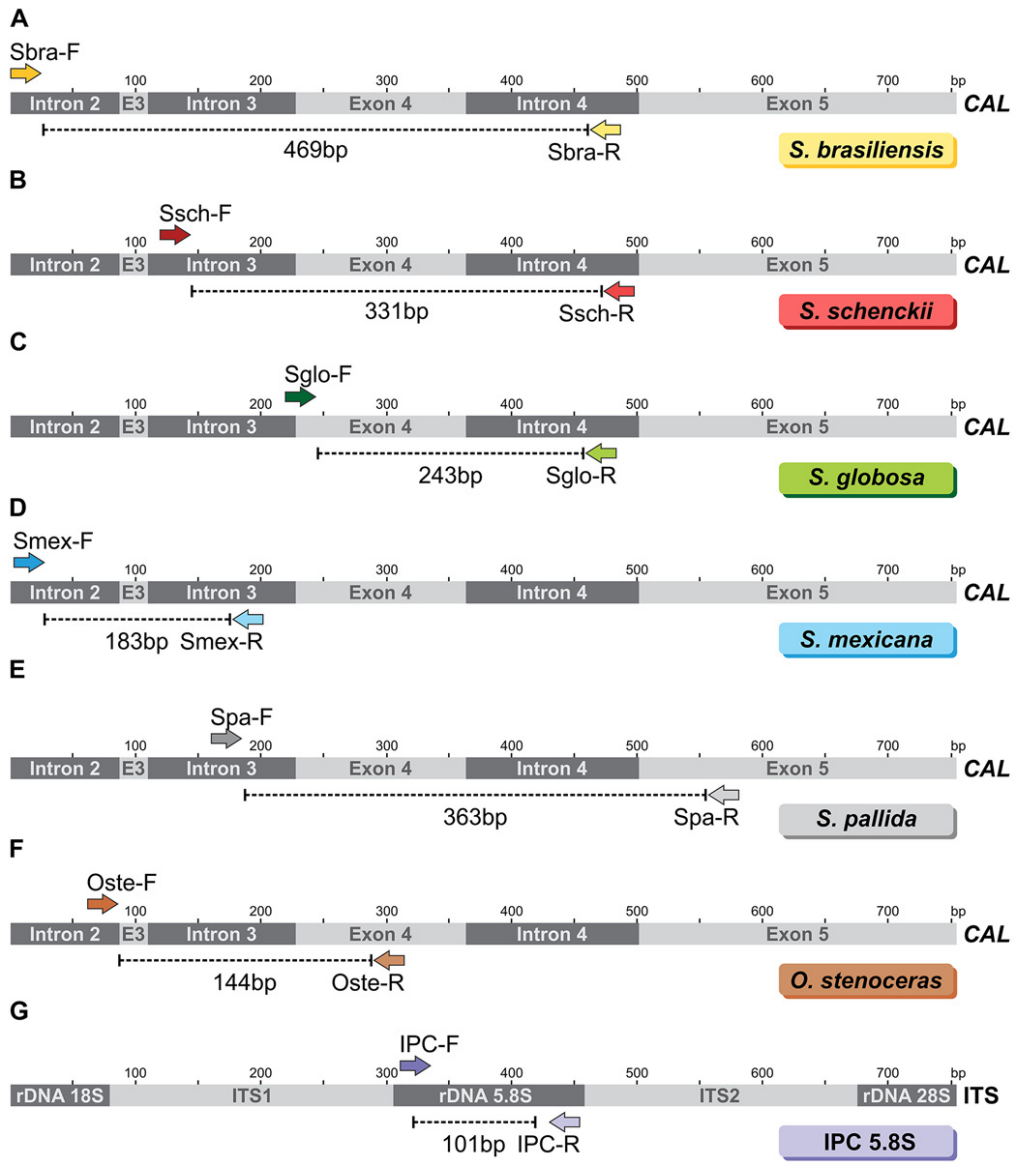
Annealing sites for species-specific primers that targeted exons 3–5 of the calmodulin gene (*CAL*), and an internal positive control (IPC) that targeted the 5.8s region of ribosomal DNA. Predicted PCR products are shown for (A) *Sporothrix brasiliensis* primers, Sbra-F and Sbra-R; (B) *Sporothrix schenckii s*. *str*. primers, Ssch-F and Ssch-R; (C) *Sporothrix globosa* primers, Sglo-F and Sglo-R; (D) *Sporothrix mexicana* primers, Smex-F and Smex-R; (E) *Sporothrix pallida* primers, Spa-F and Spa-R; (F) *Ophiostoma stenoceras* primers, Oste-F and Oste-R. Above each depiction of the *CAL* gene, a scale indicates the sequence length, with ticks at intervals of 50 bp. (G) IPC primers IPC-F and IPC-R. ITS: internal transcribed spacer

Primer design was based on the alignment of sequences from target and non-target *Sporothrix* species. The sequence database was built by combining published sequences of *CAL* from GenBank and sequences generated in our laboratory. Species-specific primer sites were found mostly in introns. Each newly designed pair of primers was tested *in silico*, and the targeted amplicons ranged between 144 and 469 bp (Fig 1 and Table 2).

**Table 2:** Species-specific primer sequences that targeted the calmodulin gene (*CAL*) in members of the genus *Sporothrix/Ophiostoma*.

Target species	Primer	Primer sequence (5′-3′)	Target	Amplicon size (bp)
*S*. *brasiliensis*	Sbra-F	CCC CCG TTT GAC GCT TGG	*CAL*	469
	Sbra-R	CCC GGA TAA CCG TGT GTC ATA AT		
*S*. *schenckii*	Ssch-F	TTT CGA ATG CGT TCG GCT GG	*CAL*	331
	Ssch-R	CTC CAG ATC ACC GTG TCA		
*S*. *globosa*	Sglo-F	CGC CTA GGC CAG ATC ACC ACT AAG	*CAL*	243
	Sglo-R	CCA ATG TCT ACC CGT GCT		
*S*. *mexicana*	Smex-F	TCT CTG CCG ACA ATT CTT TCT C	*CAL*	183
	Smex-R	GGA AAG CGG TGG CTA GAT GC		
*S*. *pallida*	Spa-F	CGC TGC TTT CCG CCA TTT TCG C	*CAL*	363
	Spa-R	GCC ATT GTT GTC GCG GTC GAA G		
*O*. *stenoceras*	Oste-F	GTG AAC ACC CTC TAT GTA CTT CG	*CAL*	144
	Oste-R	GTG TAG AGG GGG ATA GAC AGT G		
*Sporothrix/Ophiostoma*	IPC-F	ATG CGA TAC GTA ATG TGA ATT GC	5.8S	101
(IPC)	IPC-R	GAC GCT CGG ACA GGC ATG		

IPC: internal positive control.

The sequence homology of each primer was assessed with a BLASTn-search [48], and no significant homology was found with human, mouse, or microorganism sequences outside the genus of *Sporothrix*. An *in vitro* PCR assay with selected primers did not amplify DNA from other pathogenic or non-pathogenic fungi, including closely related species; this result confirmed the high specificity predicted in the BLASTn-search analyses.

### *In vitro* PCR with species-specific primers

**Fig 2:**
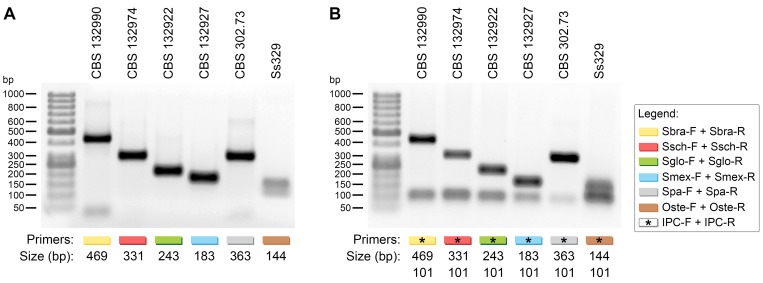
Agarose gel electrophoresis shows successful amplification with specific primer sets and templates. (A) (*Lane 1*) 50 bp DNA ladder (Fermentas, USA) for size determinations; (*Lane 2*) Sbra-F and Sbra-R with *S*. *brasiliensis* DNA (CBS 132990); (*Lane 3*) Ssch-F and Ssch-R with *S*. *schenckii s*. *str*. DNA (CBS 132974); (*Lane 4*) Sglo-F and Sglo-R with *S*. *globosa* DNA (CBS 132922); (*Lane 5*) Smex-F and Smex-R with *S*. *mexicana* DNA (CBS 132927); (*Lane 6*) Spa-F and Spa-R with *S*. *pallida* DNA (CBS 302.73); (*Lane 7*) Oste-F and Oste-R with *O*. *stenoceras* DNA (Ss329). (B) A second pair of primers was added to each reaction as an internal positive control (IPC), which targeted the 5.8s region of ribosomal DNA. IPC primers generated a 101 bp amplicon; samples that contained IPC primers are indicated with an asterisk. The absence of an IPC product indicated that PCR was inhibited. The presence of IPC product and absence of the target indicated interference from a polymorphism in the DNA target of the species-specific primers.

With 100 ng of DNA derived from *Sporothrix* species, we successfully amplified the target regions of the primer pairs, Sbra-F and Sbra-R for *S*. *brasiliensis* (469 bp); Ssch-F and Ssch-R for *S*. *schenckii* (331 bp); Sglo-F and Sglo-R for *S*. *globosa* (243 bp); Smex-F and Smex-R for *S*. *mexicana* (183 bp); Spa-F and Spa-R for *S*. *pallida* (363 bp); and Oste-F and Oste-R for *O*. *stenoceras* (144 bp) (Fig 2A). Moreover, the IPC primers, which targeted the 5.8s region of the ribosomal DNA operon, generated a 101 bp amplicon in all species (Fig 2B). Next, these species-specific primers were tested for their capabilities for identifying reference isolates [1, 3, 17, 40] that harbored DNA of the three main pathogenic agents, *S*. *brasiliensis* (n = 35), *S*. *schenckii* (n = 48), and *S*. *globosa* (n = 6). These PCR assays were in agreement (100%) with the identifications achieved with molecular phylogeny of *CAL* sequences (*Kappa* = 1.0; see S1 Table). In this study, all negative controls remained negative after PCR.

**Fig 3:**
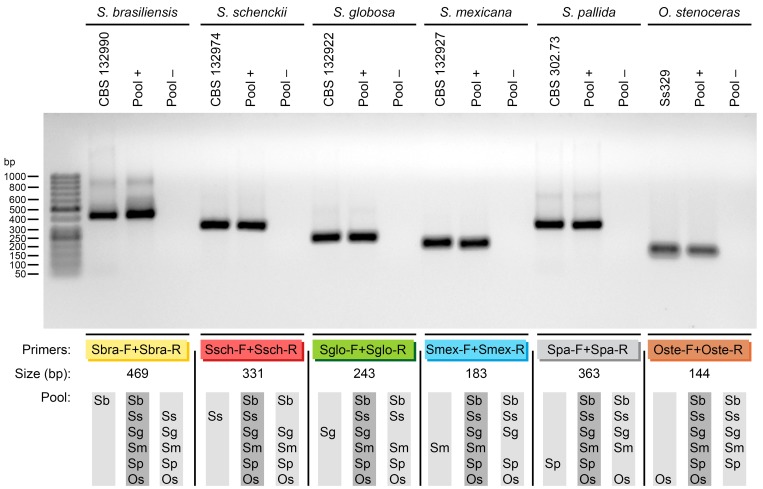
Specificity in the presence of non-target templates. A pooled DNA sample (pool +) comprised equal volumes of the following DNA templates: *S*. *brasiliensis* (Sb: CBS 120339), *S*. *schenckii s*. *str*. (Ss: CBS 359.36), *S*. *globosa* (Sg: CBS 120340), *S*. *mexicana* (Sm: CBS 120341), *S*. *pallida* (Sp: CBS 302.73), and *O*. *stenoceras* (Os: Ss329). The specificity of amplification was confirmed by removing the target DNA from the pooled sample (pool -) for each set of primers. (*Left lane*) 50 bp DNA Ladder (Fermentas, USA) for sizing the amplicons.

The specificity of amplification was evaluated with a complex pool of 5 non-target DNA templates plus a specific-target DNA template (Table 1). For example, we prepared a pool of DNA templates (20 ng of each/reaction) with the following species *S*. *brasiliensis*, *S*. *schenckii s*. *str*., *S*. *globosa*, *S*. *mexicana*, *S*. *pallida*, and *O*. *stenoceras* (pool +). We added only the Sbra-F and Sbra-R primers for the PCR. This reaction produced only the specific 469 bp fragment, similar to that obtained in the reaction with only *S*. *brasiliensis* DNA (100 ng/reaction). However, when *S*. *brasiliensis* DNA was omitted from the mixture (pool -), no product was amplified with the Sbra-F and Sbra-R primers (Fig 3).

### Analytical sensitivity

**Fig 4:**
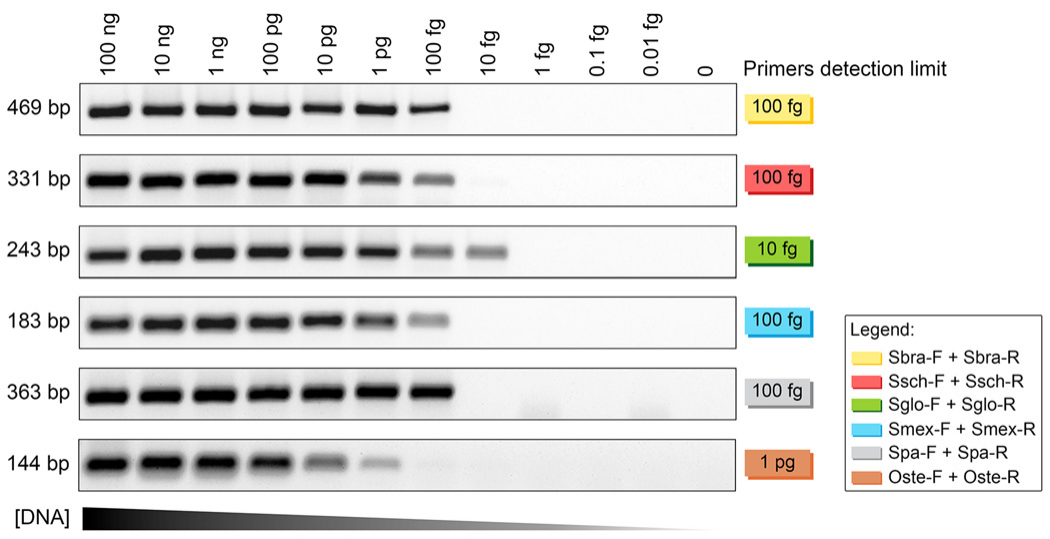
The sensitivity of primer sets for amplification tested with 10-fold serial dilutions of *Sporothrix* spp. DNA. Agarose gel electrophoresis shows different amounts of DNA templates (100 ng to 0.01 fg) amplified with the corresponding primer sets. (*Top row*) Sbra-F and Sbra-R with *S*. *brasiliensis* DNA (CBS 132990); (*row 2*) Ssch-F and Ssch-R with *S*. *schenckii s*. *str*. DNA (CBS 132974); (*row 3*) Sglo-F and Sglo-R with *S*. *globosa* DNA (CBS 132922); (*row 4*) Smex-F and Smex-R with *S*. *mexicana* DNA (CBS 132927); (*row 5*) Spa-F and Spa-R with *S*. *pallida* DNA (CBS 302.73); (*row 6*) Oste-F and Oste-R with *O*. *stenoceras* DNA (Ss329).

The analytical sensitivity of each primer pair was determined by testing amplification with a set of 10-fold serial DNA dilutions (Fig 4). The limit of DNA detection with UV-transillumination was 100 fg for *S*. *brasiliensis*, *S*. *schenckii*, *S*. *mexicana*, and *S*. *pallida* primers. The most sensitive primers could detect 10 fg of *S*. *globosa* DNA, and the least sensitive primers required 1 pg of template to detect *O*. *stenoceras*. This analytical sensitivity assay was performed with a single round of PCR.

### Molecular assay robustness

We evaluated the efficiency and consistency of the PCR assay by testing a wide range of critical parameters. No inter- or intra-assay variation was observed. The PCR band patterns remained consistent with low and high primer concentrations, with modified DNA template quantities, and with different PCR cycling parameters (extension times). Therefore, we introduced a block cycling protocol that could streamline the assay by testing all the primer pairs simultaneously. This PCR-assay proved to be highly reliable.

### *In vivo* detection of *Sporothrix* DNA

Our second objective was to ascertain whether *Sporothrix* DNA could be detected in fresh animal tissue samples with PCR. We employed a murine model of disseminated sporotrichosis in BALB/c mice. Infections were confirmed by assaying CFUs, which revealed the success rate of inoculation [19]. Mice developed several nodules and reddish swellings at the site of inoculation and along the tail. No cutaneous lesions were observed in any of the groups. However, at necropsy, animals showed pronounced swelling of visceral organs, particularly liver and spleen.

**Fig 5:**
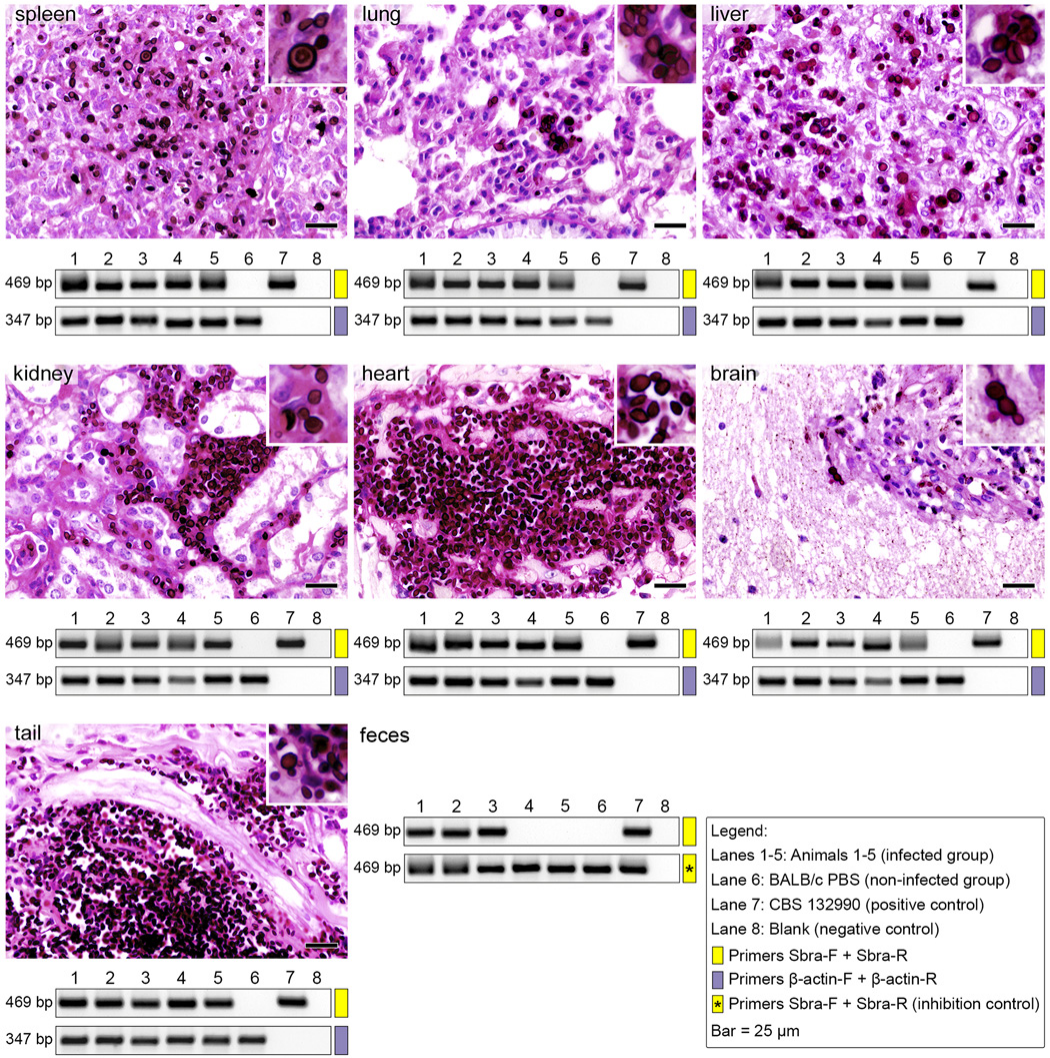
*Sporothrix brasiliensis* distribution in mouse organs at 10 days post infection. Mice were challenged with intravenous injections of 1×10^6^ yeast cells (*S*. *brasiliensis*—CBS 132990). Gel electrophoresis (1.2% agarose) show PCR products (5-μL aliquots) amplified with primers, Sbra-F and Sbra-R (469 bp; yellow). Template DNAs were extracted and purified directly from fresh tissues (n = 5) dissected from (*left-to-right*, *top-to-bottom*) spleen, lungs, liver, kidneys, heart, brain, tail, and feces. Control reactions (347 bp; purple) used primers that recognized β-actin of the mouse genome. For PCR assays of DNA extracted from feces, the inhibition control was assessed by spiking the reaction solution with genomic DNA (100 ng) extracted from a *S*. *brasiliensis* culture (CBS 132990). Statistical analyses of the diagnostic accuracies are shown in S2 Table.

**Fig 6:**
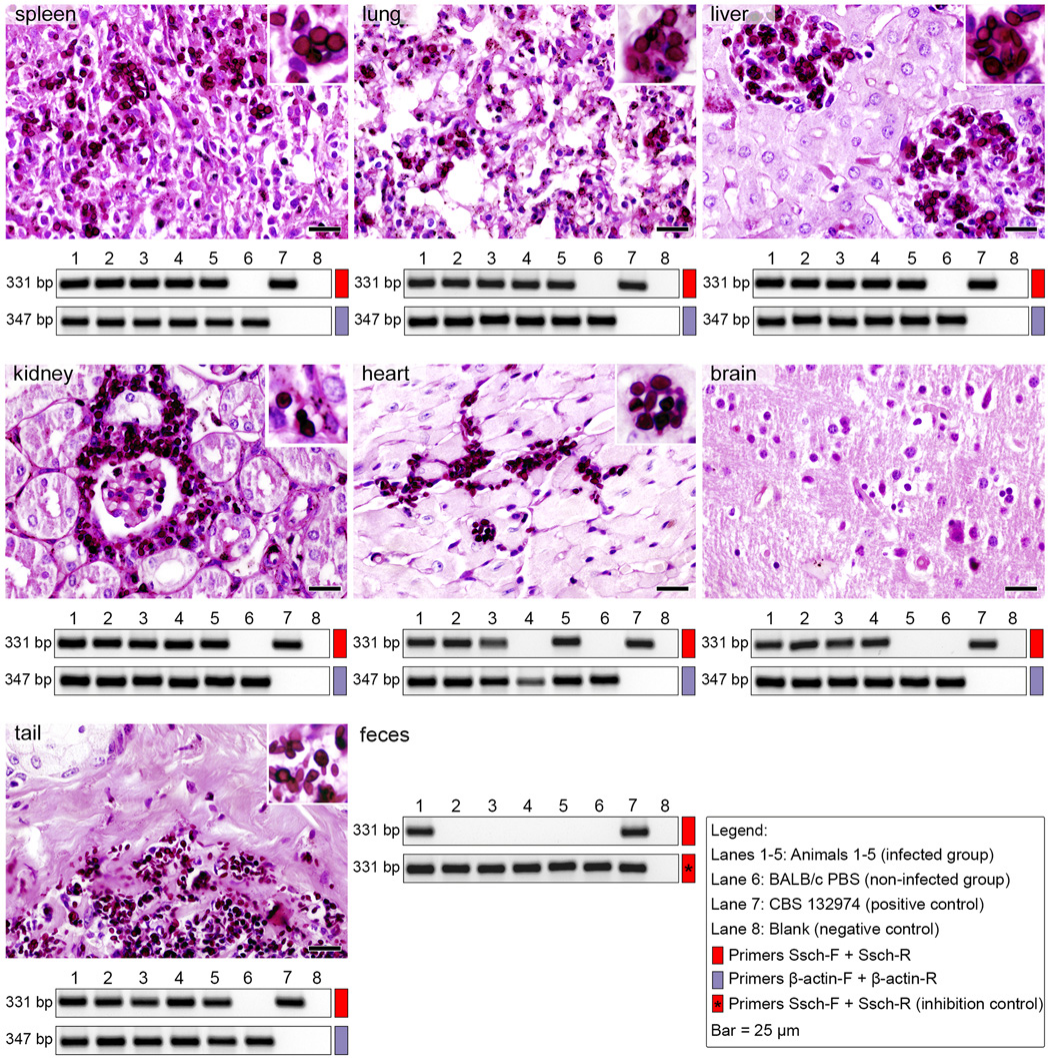
*Sporothrix schenckii* distribution in mouse organs at 10 days post infection. Mice were challenged with intravenous injections of 5×10^6^ yeast cells (*S*. *schenckii*—CBS 132974). Gel electrophoresis (1.2% agarose) show PCR products (5-μL aliquots) amplified with primers, Ssch-F and Ssch-R (331 bp; red). Template DNAs were extracted and purified directly from fresh tissues (n = 5) dissected from (*left-to-right*, *top-to-bottom*) spleen, lungs, liver, kidneys, heart, brain, tail, and feces. Control reactions (347 bp; purple) used primers that recognized β-actin of the mouse genome. For PCR assays of DNA extracted from feces, the inhibition control was assessed by spiking the reaction solution with genomic DNA (100 ng) extracted from a *S*. *schenckii* culture (CBS 132974). Statistical analyses of the diagnostic accuracies are shown in S2 Table.

PCR was performed with control primers that targeted BALB/c-mouse β-actin DNA in all three groups (two infected groups and one control group). Samples from all organs showed positive detection of β-actin DNA, indicating the absence of PCR inhibitors. The primers, Sbra-F and Sbra-R, successfully detected target DNA from the highly pathogenic strain, *S*. *brasiliensis*. Detection rates (%) were high in spleen (100%), liver (100%), lungs (100%), heart (100%), brain (100%), kidneys (100%), tail (100%), and feces (60%) samples derived from group I infected animals, but not in samples from the control group (Fig 5). Similarly, the primers, Ssch-F and Ssch-R, successfully detected *S*. *schenckii* DNA in tissue samples. Detection rates (%) were high in spleen (100%), liver (100%), lungs (100%), heart (80%), brain (80%), kidneys (100%), tail (100%), and feces (20%) samples derived from group II infected animals, but not in samples from the control group (Fig 6). Diagnostic accuracies of individual primers for *S*. *brasiliensis* and *S*. *schenckii* detection are presented in S2 Table.

Histological analysis of tissue sections from experimentally infected mice demonstrated the presence of *S*. *brasiliensis* and *S*. *schenckii* yeast cells in all evaluated organs (except brain for *S*. *schenckii*), with some differences in the fungal load per organ (CFU assay). Typically, CFU analyses showed that the main organs affected were liver, spleen, and lungs, which correlated with the high intensities observed in the PCR assays. The tissues from both infected groups (*S*. *brasiliensis* and *S*. *schenckii*) showed good agreement between the CFU and PCR assays (*Kappa* = 0.8–1.0; S2 Table), except for heart and brain of animals infected with *S*. *schenckii* (*Kappa* = 0). Histopathological analyses showed the fungus in the heart, but not the brain, of *S*. *schenckii-*infected animals. In this study, the PCR assay in some tissue specimens outperformed conventional methods, and proved to be highly sensitive (S2 Table). The PCR, culture, and histology analyses showed negative results for all uninfected mice.

Remarkably, our PCR assay detected *Sporothrix* DNA in fecal samples. This assay showed considerable differences in DNA intensities in feces samples from three *S*. *brasiliensis*-infected animals (Fig 5) and in one animal infected with *S*. *schenckii* (Fig 6). We subsequently introduced 100 ng of a purified sample of *S*. *brasiliensis* or *S*. *schenckii* DNA (spiked into the PCR reaction solution) to check for the presence of PCR inhibition. Positive amplifications suggested the absence of PCR inhibitors for both groups (inhibition control; Figs 5 and 6).

### Conidial PCR genotyping of *Sporothrix*

**Fig 7:**
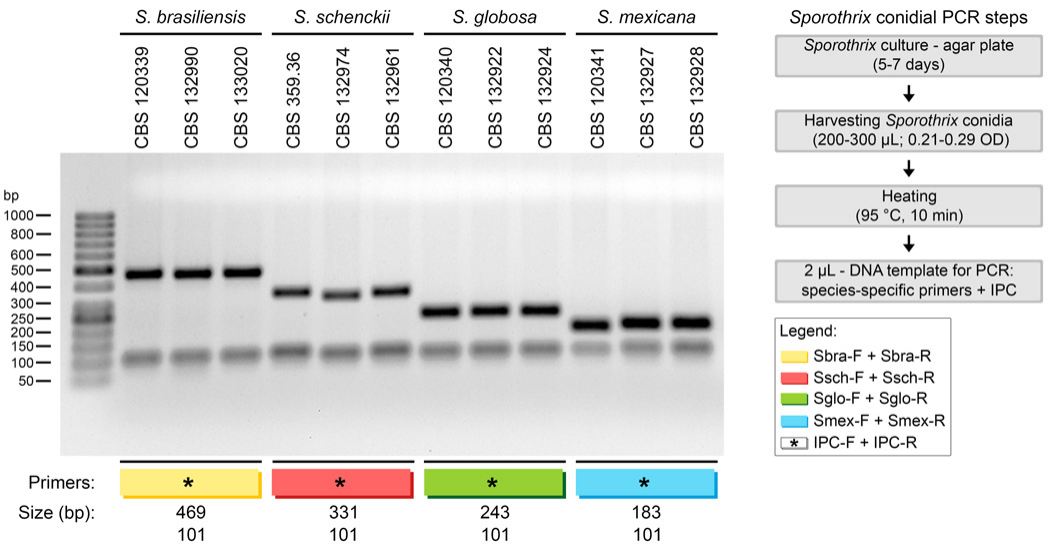
Conidial PCR amplified with species-specific primers and internal positive control (IPC) primers for genotyping *Sporothrix* spp. A representative, 1.2% agarose gel shows PCR products (5-μL aliquots) amplified from DNA derived from conidia of *S*. *brasiliensis* (CBS 120339, CBS 132990, and CBS 133020), *S*. *schenckii* (CBS 359.36, CBS 132974, and CBS 132961), *S*. *globosa* (CBS 120340, CBS 132922, and CBS 132924), and *S*. *mexicana* (CBS 120341, CBS 132927, and CBS 132928). IPC bands are 101 bp; samples that contained IPC primers are indicated with an asterisk.

In an effort to genotype *Sporothrix* spp. with conidia as a source of DNA, we used the following procedure: (1) *Sporothrix* conidia were collected from agar plates and disrupted by heating at 95°C, and (2) a 2-μl aliquot was used directly in PCR reactions. Cell disruption and the presence of DNA were checked by amplifying the IPC. The presence of specific *Sporothrix* DNA was successfully detected with species-specific primers for a large set of specimens (Fig 7).

## Discussion

Understanding the distribution patterns of cryptic *Sporothrix* species is fundamental for addressing questions concerning epidemiology and evolution. It is also a prerequisite for the development of public health programs that can tackle future outbreaks. Historically, the morphological plasticity of agents of sporotrichosis hampered identification; but this problem was alleviated when molecular techniques became routine in fungal diagnosis [2, 33]. Here, we described a new PCR assay for identification and detecting *Sporothrix* species.

Molecular tools are far more sensitive than histology for accurately detecting *Sporothrix*. The results obtained in the present study demonstrated that the PCR assay had excellent sensitivity; as little as 10 fg of *Sporothrix* DNA could be detected in a single round of PCR. Identification of 90 strains showed that the PCR assay was highly specific and rapid for identifying a wide range of medically important *Sporothrix* species. The PCR results were in very good agreement with results obtained with *CAL* phylogeny (S1 Table), which reinforces the usefulness of our tests for the rapid and accurate identification of pathogenic *Sporothrix* species. Moreover, we demonstrated that our PCR assay could specifically detect *Sporothrix* DNA, even in a complex mixture of known, closely related *Sporothrix-Ophiostoma* species. There are many versions of molecular assays, ranging from conventional PCR [52–55] to RFLP [56]. However, these methodologies were not ideal for identifying cryptic species; none of these methodologies alone or in combination were able to differentiate reliably among several species in the *S*. *schenckii* clade, especially because they were proposed before the recognition and introduction of new agents. In this scenario, the gold standard for recognizing these pathogenic entities is to analyze protein-encoding loci, such as the genes that encode calmodulin, beta-tubulin, and translation elongation factor [4, 9, 10, 36]. Compared to the other assays available for molecular identification of *Sporothrix* species, such as PCR-RFLP (usually 20,5 h) [2] and DNA sequencing (usually 10 h) [3, 4, 9, 14], our PCR assays with species-specific primers provided a more rapid, efficient approach (usually 6,5 h). Our species-specific primers assay generated results with high sensitivity and reliability; it was easy to perform; and it consistently reduced the costs of species identification.

The *CAL* gene sequences in *Sporothrix* are highly divergent in non-coding regions, but they show little variation in the protein-coding regions. In protein-coding regions, variations are typically synonymous substitutions, indicating that the intact protein is stringently conserved to preserve the original function. Despite the fact that many microorganisms, including *Sporothrix* [57], typically harbor only a single copy of *CAL* in the genome, some important points led us to choose *CAL* as a target in developing a molecular diagnostic test. Clearly, *CAL* has several parsimonious informative sites along its sequence, which demonstrate its importance in phylogenetic recognition among the species included in the *S*. *schenckii* clade [1, 10, 13, 14, 42]. Furthermore, *CAL* has been extensively used for the molecular characterization of clinical specimens [1, 3, 9, 13, 14, 17, 24, 42, 58]; therefore, a large number of sequences are available in public databases. This feature allowed us to minimize a possible bias during primer design, because we could choose parsimonious informative sites that were conserved intra-specifically. This lack of bias was further corroborated with a stringent primer specificity analysis with the Primer-BLAST program, where primers were tested against a global data set. The results strongly suggested that the primers developed here might successfully amplify isolates from all continents with epidemics of sporotrichosis [9]. We performed *in vitro* amplifications of isolates from Peru, Mexico, the USA, Spain, and Japan. The results supported our *in silico* findings (S1 Table).

We initially assessed murine sporotrichosis with histological analyses of pathognomonic structures from mouse samples. Those results were confirmed with counts of CFUs. Together, these assays presented good sensitivity for samples from spleen, liver, lungs, heart, brain, kidneys, and tails of infected animals, which confirmed successful dissemination of the fungus throughout the organs. Our subsequent molecular diagnosis of sporotrichosis had sensitivity and specificity values that approached 100%; this PCR assay outperformed histological and or/ CFU assays in the detection of *S*. *schenckii* infections in specimens from heart and brain; this result suggested that our assay may be a valuable tool that facilitates the direct diagnosis of sporotrichosis (S2 Table). In general, other studies that tested species-specific PCR for diagnosing fungal infections reported potential sensitivity and specificity values >90%, including studies in *Aspergillus* [59], *Candida* [60], *Fusarium* [61], *Talaromyces* [62], and *Paracoccidioides* [63] isolated from different clinical specimens. Our species-specific PCR assay performed well with various tissue specimens. Nevertheless, challenges associated with PCR-based fungal diagnostic assays should be considered. In particular, they lack standardized criteria for evaluating technical issues, e.g. DNA extraction, primer design, amplification, PCR inhibition controls, and contamination controls.

Animal feces may be valuable samples for examining the ecology and epidemiology of sporotrichosis [28, 64]. In implementing measures to contain the progress of an epidemic, it is important to know whether feces from diseased animals may infect adjacent soil, and thus, increase the risk of outbreaks in endemic areas. Feces are among the most complex samples for direct PCR testing, due to the presence of several PCR inhibitors, such as heme, complex carbohydrates, and particularly, bilirubins, bile salts, and phenolic compounds, which are often coextracted with pathogen DNA [65]. We removed these inhibitors with an effective DNA purification protocol, based on guanidinium thiocyanate. In addition, low amounts of DNA, resulting from incomplete cell lysis, may limit detection. We achieved physical cell lysis with rigorous homogenization by incubating feces samples on a Precellys 24 instrument. The successful detection of *Sporothrix* DNA in feces samples from experimentally infected animals demonstrated the potential of this PCR method for eco-epidemiological applications. Moreover, this finding was ecologically important, because it suggested another route for spreading infectious propagules in the environment.

Direct use of conidia as a source of genomic DNA for PCR was first proposed by Aufauvre-Brown *et al*. [66]. They demonstrated the technique with *Aspergillus* transformants. However, despite the low cost, that technique did not gain much popularity in mycology. The method has been used, with adaptations, for screening *Aspergillus* [66], *Magnaporthe* [67], *Colletotrichum* [68], and *Penicillium* [69]. We found that this method could be applied to genotyping *Sporothrix* species with the species-specific primers we developed. In an epidemic, thousands of clinical cases emerge within a short period of time; thus, the availability of a simple, fast, inexpensive method for accurately identifying the etiological agent is relevant. *Sporothrix* species produce hyaline conidia, distributed on top of conidiophores, and brown, sessile conidia that form alongside the hyphae. The amount and type of conidia produced are different among different species and isolates [38]. The advantage of the *Sporothrix* conidial PCR technique is that samples are rapidly loaded. Heat-treated conidia can be serially diluted to adjust cell density. In addition, IPC primers that target the 5.8S ribosomal region should be included to confirm the release of DNA after heating and cell disruption. In addition, the IPC can indicate whether PCR inhibitors or a polymorphism in the target regions might have interfered with amplification.

*Sporothrix* infections have been on the rise in recent years. These infections are difficult to treat, due to the lack of diagnostics, effective antifungal therapies, and vaccines. Once an outbreak occurs, especially with zoonotic involvement, rapid diagnosis is essential for minimizing further transmission. The identification method described here can be applied in outbreak areas to guide specific antifungal drug treatment of patients with sporotrichosis. The selection of an effective treatment partly relies on the correct identification of the etiological agent. With traditional methods, identification may require up to 21 days, and the results are often inconclusive. Molecular identification and diagnostics can be performed with microorganisms that are no longer viable, and free DNA can be detected from a wide range of samples, including feces.

## Supporting Information

S1 Checklist STARD checklist. (DOC)

S1 Table Strains, species, origin, and GenBank accession numbers of *Sporothrix* spp. isolates used in this study. (DOC)

S2 Table Diagnostic accuracies from individual studies for *S*. *brasiliensis* and *S*. *schenckii* murine infections compared between two assays: polymerase chain reaction (PCR) and colony-forming unit (CFU) counts, for detecting the pathogen. (DOC)

S1 Flow Diagram STARD S1 flow diagram. (PDF)
