# Supplementary material for: Molecular Diagnosis of Pathogenic Sporothrix Species
Source: PLoS Negl Trop Dis. 2015 Dec 1;9(12):e0004190. doi: 10.1371/journal.pntd.0004190 (PMC4666615; doi:10.1371/journal.pntd.0004190)
Supplement: S1 Table — (DOC) [file pntd.0004190.s002.doc]

**S1 Table. Strains, species, origin, and GenBank accession numbers of *Sporothrix* spp. isolates used in this study.**

| **Isolate** | **CBS code** | **Species** | | **Source** | **Origin** | **GenBank accession**  **number (*CAL*)** | **Reference** |
| --- | --- | --- | --- | --- | --- | --- | --- |
|  |  | ***CAL*-Phylogeny^1^** | **PCR^2^** |  |  |  |  |
| Ss05 | CBS 132985 | *S. brasiliensis* | *S. brasiliensis* | Feline sporotrichosis | Minas Gerais, Brazil | KC693830 | [1] |
| Ss07 | CBS 132986 | *S. brasiliensis* | *S. brasiliensis* | Human sporotrichosis | Minas Gerais, Brazil | KC693831 | [1] |
| Ss08 | - | *S. brasiliensis* | *S. brasiliensis* | Human sporotrichosis | Minas Gerais, Brazil | KC693832 | [1] |
| Ss09 | - | *S. brasiliensis* | *S. brasiliensis* | Human sporotrichosis | Minas Gerais, Brazil | KC693833 | [1] |
| Ss10 | CBS 132987 | *S. brasiliensis* | *S. brasiliensis* | Human sporotrichosis | Minas Gerais, Brazil | KC693834 | [1] |
| Ss14 | - | *S. brasiliensis* | *S. brasiliensis* | Human sporotrichosis | Minas Gerais, Brazil | KF943632 | [2] |
| Ss25 | CBS 132988 | *S. brasiliensis* | *S. brasiliensis* | Human sporotrichosis | Paraná, Brazil | KC693840 | [1] |
| Ss27 | - | *S. brasiliensis* | *S. brasiliensis* | Human sporotrichosis | Paraná, Brazil | JX077111 | [3] |
| Ss32 | - | *S. brasiliensis* | *S. brasiliensis* | Human sporotrichosis | Paraná, Brazil | KF943636 | [2] |
| Ss33 | - | *S. brasiliensis* | *S. brasiliensis* | Human sporotrichosis | Paraná, Brazil | KF943637 | [2] |
| Ss38 | - | *S. brasiliensis* | *S. brasiliensis* | Human sporotrichosis | Paraná, Brazil | KC693844 | [1] |
| Ss43 | - | *S. brasiliensis* | *S. brasiliensis* | Human sporotrichosis | Ceará, Brazil | JX077112 | [3] |
| Ss44 | - | *S. brasiliensis* | *S. brasiliensis* | Human sporotrichosis | Ceará, Brazil | KF943641 | [2] |
| Ss52 | - | *S. brasiliensis* | *S. brasiliensis* | Human sporotrichosis | São Paulo, Brazil | KC693845 | [1] |
| Ss53 | CBS 132989 | *S. brasiliensis* | *S. brasiliensis* | Feline sporotrichosis | Rio Grande do Sul, Brazil | KC693846 | [1] |
| Ss54 | CBS 132990 | *S. brasiliensis* | *S. brasiliensis* | Feline sporotrichosis | Rio Grande do Sul, Brazil | JQ041903 | [3] |
| Ss55 | - | *S. brasiliensis* | *S. brasiliensis* | Human sporotrichosis | Rio Grande do Sul, Brazil | KC693847 | [1] |
| Ss56 | - | *S. brasiliensis* | *S. brasiliensis* | Human sporotrichosis | Rio Grande do Sul, Brazil | KC693848 | [1] |
| Ss57 | - | *S. brasiliensis* | *S. brasiliensis* | Human sporotrichosis | Rio Grande do Sul, Brazil | KF943645 | [2] |
| Ss62 | CBS 132991 | *S. brasiliensis* | *S. brasiliensis* | Human sporotrichosis | Espírito Santo, Brazil | JX077113 | [3] |
| Ss70 | - | *S. brasiliensis* | *S. brasiliensis* | Human sporotrichosis | Rio de Janeiro, Brazil | KC693850 | [1] |
| Ss71 | - | *S. brasiliensis* | *S. brasiliensis* | Human sporotrichosis | Rio de Janeiro, Brazil | KC693851 | [1] |
| Ss72 | - | *S. brasiliensis* | *S. brasiliensis* | Human sporotrichosis | Rio de Janeiro, Brazil | KC693852 | [1] |
| Ss74 | - | *S. brasiliensis* | *S. brasiliensis* | Human sporotrichosis | Rio de Janeiro, Brazil | KF943651 | [2] |
| Ss76 | - | *S. brasiliensis* | *S. brasiliensis* | Human sporotrichosis | Rio de Janeiro, Brazil | KF943652 | [2] |
| Ss77 | - | *S. brasiliensis* | *S. brasiliensis* | Human sporotrichosis | Rio de Janeiro, Brazil | KF943653 | [2] |
| **Isolate** | **CBS code** | **Species** | | **Source** | **Origin** | **GenBank accession**  **number (*CAL*)** | **Reference** |
|  |  | ***CAL*-Phylogeny^1^** | **PCR^2^** |  |  |  |  |
| Ss150 | - | *S. brasiliensis* | *S. brasiliensis* | Human sporotrichosis | Rio Grande do Sul, Brazil | KC693863 | [1] |
| Ss151 | CBS 132994 | *S. brasiliensis* | *S. brasiliensis* | Canine sporotrichosis | Rio Grande do Sul, Brazil | KC693864 | [1] |
| Ss152 | CBS 132995 | *S. brasiliensis* | *S. brasiliensis* | Feline sporotrichosis | Rio Grande do Sul, Brazil | KC693865 | [1] |
| Ss153 | CBS 132996 | *S. brasiliensis* | *S. brasiliensis* | Feline sporotrichosis | Rio Grande do Sul, Brazil | KC693866 | [1] |
| Ss154 | - | *S. brasiliensis* | *S. brasiliensis* | Feline sporotrichosis | Rio Grande do Sul, Brazil | KC693867 | [1] |
| Ss155 | - | *S. brasiliensis* | *S. brasiliensis* | Feline sporotrichosis | Rio Grande do Sul, Brazil | KC693868 | [1] |
| Ss260 | CBS 133019 | *S. brasiliensis* | *S. brasiliensis* | Feline sporotrichosis | Rio Grande do Sul, Brazil | KC693893 | [1] |
| Ss261 | - | *S. brasiliensis* | *S. brasiliensis* | Human sporotrichosis | Rio Grande do Sul, Brazil | KC693894 | [1] |
| Ss265 | CBS 133020 | *S. brasiliensis* | *S. brasiliensis* | Human sporotrichosis | Minas Gerais, Brazil | JN204360 | [4] |
| Ss06 | CBS 132922 | *S. globosa* | *S. globosa* | Human sporotrichosis | Minas Gerais, Brazil | JF811336 | [3] |
| Ss41 | CBS 132923 | *S. globosa* | *S. globosa* | Human sporotrichosis | Ceará, Brazil | JF811337 | [3] |
| Ss49 | CBS 132924 | *S. globosa* | *S. globosa* | Human sporotrichosis | Goiás, Brazil | JF811338 | [3] |
| Ss236 | CBS 132925 | *S. globosa* | *S. globosa* | Human sporotrichosis | Minas Gerais, Brazil | KC693877 | [1] |
| FMR 8600 | CBS 120340 | *S. globosa* | *S. globosa* | Human sporotrichosis | Spain | AM116908 | [5] |
| FMR8595 | CBS 130104 | *S. globosa* | *S. globosa* | Human sporotrichosis | Spain | AM116905 | [5] |
| Ss01 | CBS 132961 | *S. schenckii* | *S. schenckii* | Feline sporotrichosis | São Paulo, Brazil | KC693828 | [1] |
| Ss02 | CBS 132962 | *S. schenckii* | *S. schenckii* | Human sporotrichosis | Rio Grande do Sul, Brazil | KC693829 | [1] |
| Ss03 | CBS 132963 | *S. schenckii* | *S. schenckii* | Human sporotrichosis | Rio Grande do Sul, Brazil | JX077117 | [3] |
| Ss04 | - | *S. schenckii* | *S. schenckii* | Human sporotrichosis | Rio Grande do Sul, Brazil | JX077118 | [3] |
| Ss13 | - | *S. schenckii* | *S. schenckii* | Human sporotrichosis | Minas Gerais, Brazil | KC693836 | [1] |
| Ss15 | - | *S. schenckii* | *S. schenckii* | Human sporotrichosis | Minas Gerais, Brazil | KC693837 | [1] |
| Ss19 | - | *S. schenckii* | *S. schenckii* | Human sporotrichosis | Paraná, Brazil | KF943633 | [2] |
| Ss20 | - | *S. schenckii* | *S. schenckii* | Human sporotrichosis | Paraná, Brazil | JX077119 | [3] |
| Ss21 | - | *S. schenckii* | *S. schenckii* | Human sporotrichosis | Paraná, Brazil | JX077120 | [3] |
| Ss22 | CBS 132964 | *S. schenckii* | *S. schenckii* | Human sporotrichosis | Paraná, Brazil | KF943634 | [2] |
| Ss26 | CBS 132965 | *S. schenckii* | *S. schenckii* | Human sporotrichosis | Paraná, Brazil | KC693841 | [1] |
| Ss39 | - | *S. schenckii* | *S. schenckii* | Human sporotrichosis | Paraná, Brazil | JQ041899 | [6] |
| Ss40 | - | *S. schenckii* | *S. schenckii* | Human sporotrichosis | Ceará, Brazil | JQ041900 | [6] |
| **Isolate** | **CBS code** | **Species** | | **Source** | **Origin** | **GenBank accession**  **number (*CAL*)** | **Reference** |
|  |  | ***CAL*-Phylogeny^1^** | **PCR^2^** |  |  |  |  |
| Ss42 | CBS 132966 | *S. schenckii* | *S. schenckii* | Human sporotrichosis | Ceará, Brazil | KF943640 | [2] |
| Ss45 | - | *S. schenckii* | *S. schenckii* | Human sporotrichosis | Goiás, Brazil | KJ020358 | [2] |
| Ss46 | - | *S. schenckii* | *S. schenckii* | Human sporotrichosis | Goiás, Brazil | KF943642 | [2] |
| Ss47 | - | *S. schenckii* | *S. schenckii* | Human sporotrichosis | Goiás, Brazil | JQ041901 | [6] |
| Ss48 | - | *S. schenckii* | *S. schenckii* | Human sporotrichosis | Goiás, Brazil | KF943643 | [2] |
| Ss50 | - | *S. schenckii* | *S. schenckii* | Human sporotrichosis | Goiás, Brazil | KF943644 | [2] |
| Ss60 | - | *S. schenckii* | *S. schenckii* | Human sporotrichosis | São Paulo, Brazil | KF943648 | [2] |
| Ss61 | - | *S. schenckii* | *S. schenckii* | Soil | São Paulo, Brazil | KF561244 | [7] |
| Ss63 | CBS 132968 | *S. schenckii* | *S. schenckii* | Human sporotrichosis | Espírito Santo, Brazil | JX077123 | [3] |
| Ss64 | - | *S. schenckii* | *S. schenckii* | Human sporotrichosis | Espírito Santo, Brazil | JX077124 | [3] |
| Ss73 | - | *S. schenckii* | *S. schenckii* | Human sporotrichosis | Rio de Janeiro, Brazil | KC693853 | [1] |
| Ss75 | - | *S. schenckii* | *S. schenckii* | Human sporotrichosis | Rio de Janeiro, Brazil | KC693854 | [1] |
| Ss78 | - | *S. schenckii* | *S. schenckii* | Human sporotrichosis | Rio de Janeiro, Brazil | KC693855 | [1] |
| Ss80 | CBS 132969 | *S. schenckii* | *S. schenckii* | Human sporotrichosis | Rio de Janeiro, Brazil | JX077125 | [3] |
| Ss90 | - | *S. schenckii* | *S. schenckii* | Human sporotrichosis | Rio de Janeiro, Brazil | KC693859 | [1] |
| Ss102 | CBS 132970 | *S. schenckii* | *S. schenckii* | Human sporotrichosis | São Paulo, Brazil | KF943671 | [2] |
| Ss103 | - | *S. schenckii* | *S. schenckii* | Human sporotrichosis | São Paulo, Brazil | KF943672 | [2] |
| Ss111 | CBS 132971 | *S. schenckii* | *S. schenckii* | Human sporotrichosis | São Paulo, Brazil | KC693860 | [1] |
| Ss113 | CBS 132972 | *S. schenckii* | *S. schenckii* | Human sporotrichosis | São Paulo, Brazil | KF943679 | [2] |
| Ss116 | - | *S. schenckii* | *S. schenckii* | Human sporotrichosis | São Paulo, Brazil | KF943680 | [2] |
| Ss117 | - | *S. schenckii* | *S. schenckii* | Human sporotrichosis | São Paulo, Brazil | KF943681 | [2] |
| Ss118 | CBS 132974 | *S. schenckii* | *S. schenckii* | Human sporotrichosis | São Paulo, Brazil | JX077126 | [3] |
| Ss124 | - | *S. schenckii* | *S. schenckii* | Human sporotrichosis | São Paulo, Brazil | KF943687 | [2] |
| Ss126 | - | *S. schenckii* | *S. schenckii* | Human sporotrichosis | São Paulo, Brazil | JQ041904 | [6] |
| Ss127 | - | *S. schenckii* | *S. schenckii* | Human sporotrichosis | São Paulo, Brazil | KF943688 | [2] |
| Ss130 | - | *S. schenckii* | *S. schenckii* | Human sporotrichosis | Pernambuco, Brazil | KF943690 | [2] |
| Ss159 | CBS 132976 | *S. schenckii* | *S. schenckii* | Human sporotrichosis | Japan | KF574464 | [7] |
| Ss160 | - | *S. schenckii* | *S. schenckii* | Human sporotrichosis | Mexico | KF574465 | [7] |
| **Isolate** | **CBS code** | **Species** | | **Source** | **Origin** | **GenBank accession**  **number (*CAL*)** | **Reference** |
|  |  | ***CAL*-Phylogeny^1^** | **PCR^2^** |  |  |  |  |
| Ss160 | - | *S. schenckii* | *S. schenckii* | Human sporotrichosis | Mexico | KF574465 | [7] |
| Ss161 | - | *S. schenckii* | *S. schenckii* | Human sporotrichosis | Mexico | KF574466 | [7] |
| Ss162 | CBS 132977 | *S. schenckii* | *S. schenckii* | Vegetal | Mexico | KF574467 | [7] |
| Ss163 | - | *S. schenckii* | *S. schenckii* | Human sporotrichosis | Peru | KF574468 | [7] |
| Ss164 | - | *S. schenckii* | *S. schenckii* | Human sporotrichosis | Peru | KF574469 | [7] |
| Ss167 | CBS 132978 | *S. schenckii* | *S. schenckii* | Soil | Peru | KF943708 | [2] |
| Ss168 | CBS 132979 | *S. schenckii* | *S. schenckii* | Human sporotrichosis | Peru | KF943709 | [2] |
| ATCC 4821 | CBS 132984 | *S. schenckii* | *S. schenckii* | Human sporotrichosis | USA | KF574470 | [7] |

^1^Phylogenetic analysis based on partial nucleotide sequences of the calmodulin encoding gene (exons 3-5); ^2^Species identification based on species-specific PCR, as proposed in this study.

**References**

1. Rodrigues AM, de Melo Teixeira M, de Hoog GS, Schubach TMP, Pereira SA, Fernandes GF, et al. Phylogenetic analysis reveals a high prevalence of *Sporothrix brasiliensis* in feline sporotrichosis outbreaks. PLoS Negl Trop Dis. 2013;7(6):e2281. doi: 10.1371/journal.pntd.0002281. PubMed PMID: 23818999; PubMed Central PMCID: PMC3688539.

2. Rodrigues AM, de Hoog GS, Zhang Y, Camargo ZP. Emerging sporotrichosis is driven by clonal and recombinant *Sporothrix* species. Emerg Microbes Infect. 2014;3(5):e32. doi: 10.1038/emi.2014.33. PubMed PMID: 26038739; PubMed Central PMCID: PMC4051365.

3. Rodrigues AM, de Hoog S, de Camargo ZP. Emergence of pathogenicity in the *Sporothrix schenckii* complex. Med Mycol. 2013;51(4):405-12. doi: 10.3109/13693786.2012.719648. PubMed PMID: 22989196.

4. Silva-Vergara ML, de Camargo ZP, Silva PF, Abdalla MR, Sgarbieri RN, Rodrigues AM, et al. Disseminated *Sporothrix brasiliensis* infection with endocardial and ocular involvement in an HIV-infected patient. Am J Trop Med Hyg. 2012;86(3):477-80. doi: 10.4269/ajtmh.2012.11-0441. PubMed PMID: 22403321; PubMed Central PMCID: PMC3284366.

5. Marimon R, Cano J, Gené J, Sutton DA, Kawasaki M, Guarro J. *Sporothrix brasiliensis*, *S. globosa*, and *S. mexicana*, three new *Sporothrix* species of clinical interest. J Clin Microbiol. 2007;45(10):3198-206. doi: 10.1128/JCM.00808-07. PubMed PMID: 17687013; PubMed Central PMCID: PMC2045377.

6. Fernandes GF, dos Santos PO, Rodrigues AM, Sasaki AA, Burger E, de Camargo ZP. Characterization of virulence profile, protein secretion and immunogenicity of different *Sporothrix schenckii sensu stricto* isolates compared with *S. globosa* and *S. brasiliensis* species. Virulence. 2013;4(3):241-9. doi: 10.4161/viru.23112. PubMed PMID: 23324498.

7. Sasaki AA, Fernandes GF, Rodrigues AM, Lima FM, Marini MM, dos S. Feitosa L, et al. Chromosomal polymorphism in the *Sporothrix schenckii* complex. PLoS One. 2014;9(1):e86819. doi: 10.1371/journal.pone.0086819. PubMed PMID: 24466257; PubMed Central PMCID: PMC3900657.
