# Supplementary material for: Molecular Diagnosis of Pathogenic Sporothrix Species
Source: PLoS Negl Trop Dis. 2015 Dec 1;9(12):e0004190. doi: 10.1371/journal.pntd.0004190 (PMC4666615; doi:10.1371/journal.pntd.0004190)
Supplement: S2 Table — (DOC) [file pntd.0004190.s003.doc]

**S2 Table. Diagnostic accuracies from individual studies for *S. brasiliensis* and *S. schenckii*** murine infections compared between two assays: polymerase chain reaction (PCR) and colony-forming unit (CFU) counts, for detecting the pathogen.

| **Species** | **Sample** | **Spleen** |  | **Lungs** |  | **Liver** |  | **Kidneys** |  | **Heart** |  | **Brain** |  | **Tail** |  | **Feces*** |
| --- | --- | --- | --- | --- | --- | --- | --- | --- | --- | --- | --- | --- | --- | --- | --- | --- |
|  | **Methods** | **PCR** | **CFU** | **PCR** | **CFU** | **PCR** | **CFU** | **PCR** | **CFU** | **PCR** | **CFU** | **PCR** | **CFU** | **PCR** | **CFU** | **PCR** |
| ***S. brasiliensis*** | AUC | 1.000±0.0 | 1.000±0.0 | 1.000±0.0 | 1.000±0.0 | 1.000±0.0 | 1.000±0.0 | 1.000±0.0 | 1.000±0.0 | 1.000±0.0 | 1.000±0.0 | 1.000±0.0 | 1.000±0.0 | 1.000±0.0 | 1.000±0.0 | 0.800±0.122 |
|  | 95% CI | 0.692–1 | 0.692–1 | 0.692–1 | 0.692–1 | 0.692–1 | 0.692–1 | 0.692–1 | 0.692–1 | 0.692–1 | 0.692–1 | 0.692–1 | 0.692–1 | 0.692–1 | 0.692–1 | 0.444–0.975 |
|  | *P* | <0.0001 | <0.0001 | <0.0001 | <0.0001 | <0.0001 | <0.0001 | <0.0001 | <0.0001 | <0.0001 | <0.0001 | <0.0001 | <0.0001 | <0.0001 | <0.0001 | 0.0143 |
|  | Sensitivity | 100% | 100% | 100% | 100% | 100% | 100% | 100% | 100% | 100% | 100% | 100% | 100% | 100% | 100% | 60% |
|  | 95% CI | 47.8–100 | 47.8–100 | 47.8–100 | 47.8–100 | 47.8–100 | 47.8–100 | 47.8–100 | 47.8–100 | 47.8–100 | 47.8–100 | 47.8–100 | 47.8–100 | 47.8–100 | 47.8–100 | 14.7–94.7 |
|  | Specificity | 100% | 100% | 100% | 100% | 100% | 100% | 100% | 100% | 100% | 100% | 100% | 100% | 100% | 100% | 100% |
|  | 95% CI | 47.8–100 | 47.8–100 | 47.8–100 | 47.8–100 | 47.8–100 | 47.8–100 | 47.8–100 | 47.8–100 | 47.8–100 | 47.8–100 | 47.8–100 | 47.8–100 | 47.8–100 | 47.8–100 | 47.8–100 |
|  | PPV | 100% | 100% | 100% | 100% | 100% | 100% | 100% | 100% | 100% | 100% | 100% | 100% | 100% | 100% | 100% |
|  | PNV | 100% | 100% | 100% | 100% | 100% | 100% | 100% | 100% | 100% | 100% | 100% | 100% | 100% | 100% | 71.43% |
|  | *Kappa* | 1.0 |  | 1.0 |  | 1.0 |  | 1.0 |  | 1.0 |  | 1.0 |  | 1.0 |  | – |
| ***S. schenckii*** | AUC | 1.000±0.0 | 1.000±0.0 | 1.000±0.0 | 0.900±0.1 | 1.000±0.0 | 1.000±0.0 | 1.000±0.0 | 1.000±0.0 | 0.900±0.1 | 0.500±0.0 | 0.900±0.1 | 0.500±0.0 | 1.000±0.0 | 0.900±0.1 | 0.600±0.100 |
|  | 95% CI | 0.692–1 | 0.692–1 | 0.692–1 | 0.555–0.997 | 0.692–1 | 0.692–1 | 0.692–1 | 0.692–1 | 0.555–0.997 | 0.187–0.813 | 0.555–0.997 | 0.187–0.813 | 0.692–1 | 0.555–0.997 | 0.262–0.878 |
|  | *P* | <0.0001 | <0.0001 | <0.0001 | 0.0001 | <0.0001 | <0.0001 | <0.0001 | <0.0001 | 0.0001 | 1.000 | 0.0001 | 1.000 | <0.0001 | 0.0001 | 0.3173 |
|  | Sensitivity | 100% | 100% | 100% | 80% | 100% | 100% | 100% | 100% | 80% | 0% | 80% | 0% | 100% | 80% | 20% |
|  | 95% CI | 47.8–100 | 47.8–100 | 47.8–100 | 28.5–99.5 | 47.8–100 | 47.8–100 | 47.8–100 | 47.8–100 | 28.4–99.5 | 0.0–52.2 | 28.4–99.5 | 0.0–52.2 | 47.8–100 | 28.5–99.5 | 0.5–71.6 |
|  | Specificity | 100% | 100% | 100% | 100% | 100% | 100% | 100% | 100% | 100% | 100% | 100% | 100% | 100% | 100% | 100% |
|  | 95% CI | 47.8–100 | 47.8–100 | 47.8–100 | 47.8–100 | 47.8–100 | 47.8–100 | 47.8–100 | 47.8–100 | 47.8–100 | 47.8–100 | 47.8–100 | 47.8–100 | 47.8–100 | 47.8–100 | 47.8–100 |
|  | PPV | 100% | 100% | 100% | 100% | 100% | 100% | 100% | 100% | 100% | – | 100% | – | 100% | 100% | 100% |
|  | PNV | 100% | 100% | 100% | 83% | 100% | 100% | 100% | 100% | 83.33% | – | 83.33% | – | 100% | 83% | 55.56% |
|  | *Kappa* | 1.0 |  | 0.8 |  | 1.0 |  | 1.0 |  | 0 |  | 0 |  | 0.8 |  | – |

PCR: polymerase chain reaction; CFU: colony-forming unit; AUC: Area under curve; 95% CI: 95% confidence interval; PPV: positive predictive value; PNV: negative predictive value; *Kappa*: agreement between PCR and CFU using *Kappa* statistic.

*BALB/c mouse feces were not evaluated with the CFU assay, due to the presence of rapidly-growing bacteria in fecal microflora.

Diagnostic values divergent are highlighted in yellow for *S. brasiliensis* and in red for *S. schenckii*.
