# Supplementary figures and images for: Molecular Diagnosis of Pathogenic Sporothrix Species
Source: PLoS Negl Trop Dis. 2015 Dec 1;9(12):e0004190. doi: 10.1371/journal.pntd.0004190 (PMC4666615; doi:10.1371/journal.pntd.0004190)

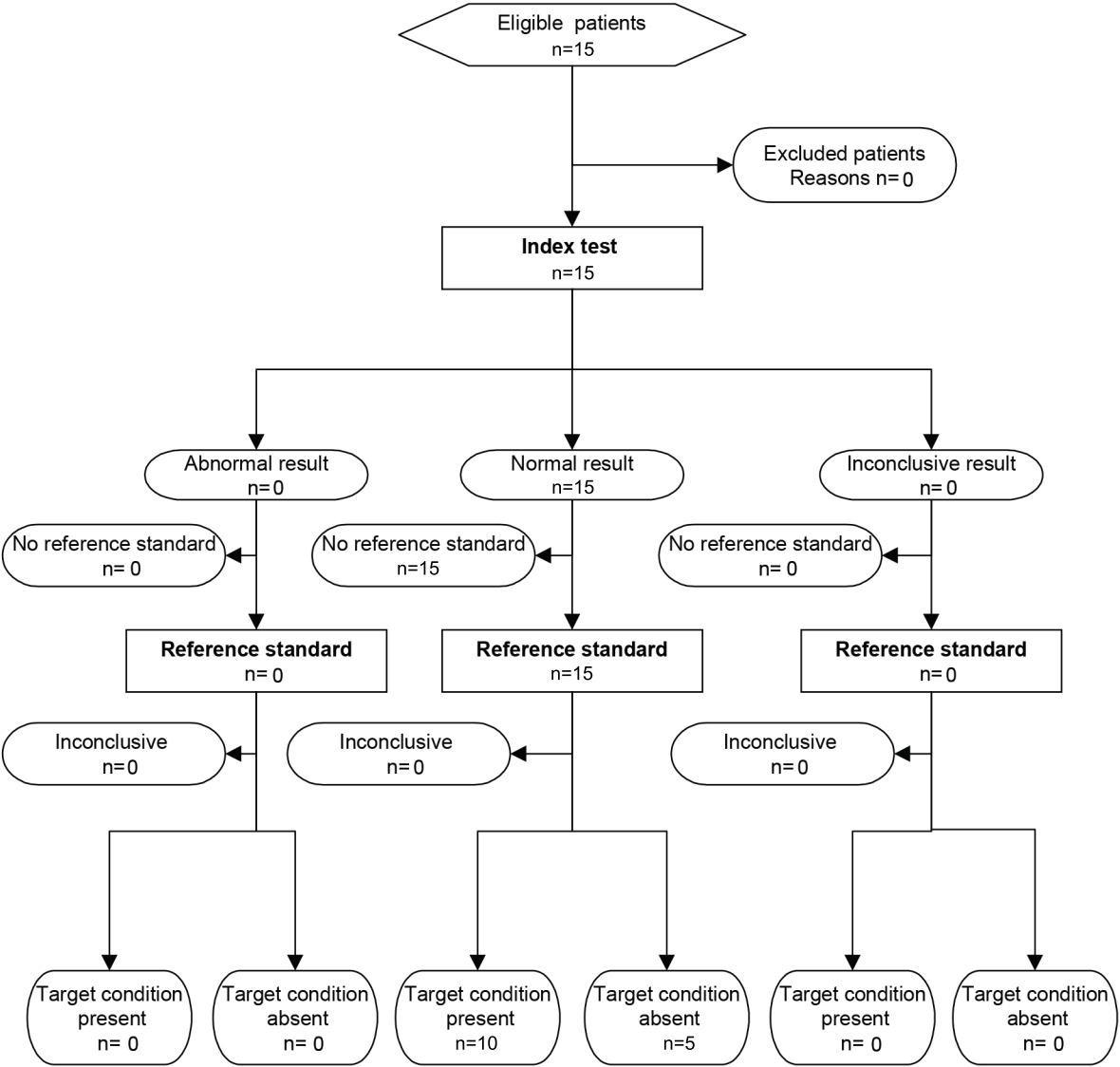

Supplement: S1 Flow Diagram — (PDF) [file pntd.0004190.s004.pdf]
